# Pulmonary Actinomycosis Mimicking Lung Cancer: A Case Report of a Rare Imitator

**DOI:** 10.7759/cureus.91553

**Published:** 2025-09-03

**Authors:** Aimen Ayaz, Salman Majeed, Suhaila Mendes, Ayesha Kazi

**Affiliations:** 1 Respiratory Medicine, Sandwell and West Birmingham Hospitals NHS Trust, Birmingham, GBR; 2 Internal Medicine, University Hospitals of Leicester NHS Trust, Leicester, GBR; 3 Internal Medicine, Sandwell and West Birmingham Hospitals NHS Trust, Birmingham, GBR

**Keywords:** antibiotics, granulomatous infection, lung cancer, positron-emission tomography (pet) avidity, pulmonary actinomycosis, pulmonary nodules

## Abstract

Pulmonary nodules mimicking lung cancer pose a significant diagnostic challenge due to overlapping clinical and radiological features, which frequently lead to referrals to chest clinics for further evaluation. A 73-year-old man presented to the chest clinic with a two-month history of fatigue, loss of appetite, and unintentional weight loss. Computed tomography (CT) scan of the chest revealed suspicious soft tissue lesions in the left upper and right middle lobes of the lungs. His medical history was notable for colorectal adenocarcinoma, treated with a right hemicolectomy in 2012, followed by adjuvant chemotherapy in 2018. A diagnostic dilemma arose when colonoscopy was inconclusive, and both pulmonary nodules demonstrated increased fluorodeoxyglucose uptake on positron-emission tomography scan, suggestive of lung malignancy. However, an initial CT-guided biopsy of the left upper lobe pulmonary nodule was nondiagnostic, and there was no evidence of recurrence of colorectal carcinoma. The diagnosis was ultimately established following a wedge resection of the right middle lobe pulmonary nodule, with histopathological analysis confirming pulmonary actinomycosis. The pulmonary nodules completely resolved after a prolonged course of antibiotics. Pulmonary actinomycosis is a rare granulomatous infection caused by the gram-positive anaerobe Actinomyces spp. Diagnosis is often delayed due to its uncommon presentation as pulmonary nodules and its ability to mimic malignancy. This case highlights the importance of including pulmonary actinomycosis in the differential diagnosis of pulmonary nodules to avoid delays in appropriate treatment.

## Introduction

Actinomycosis is a chronic bacterial infection that is often diagnosed late or misdiagnosed due to several factors, primarily its low incidence and slow progression. This rare infection is caused by bacteria of the genus Actinomyces, mainly *Actinomyces israelii*, which are normally commensal organisms in the colon, oral cavity, and vagina. When these bacteria become virulent, they can cause infections in these regions, which are classified into cervicofacial, abdominal, and pelvic actinomycosis [1].

Bacterial invasion is characterized by penetration and suppuration into submucosal tissue layers, followed by mucosal breach, and leads to the formation of abscesses and granulomas [2]. Studies have shown that any procedure disrupting the mucosal barrier, such as surgery or endoscopy, can facilitate the spread of this bacterial infection. Additionally, a weakened immune system is a significant contributing factor [3]. Heavy smoking and recurrent aspiration are important predisposing factors for pulmonary involvement [4]. This case report summarizes an atypical presentation, radiological features that overlap with malignancy, the diagnostic workup, and the therapeutic approach that led to favorable outcomes.

This case was presented as an oral presentation at the Midland Thoracic Society Autumn Meeting, Birmingham, United Kingdom, on November 8, 2024.

## Case presentation

A 73-year-old man was referred to the chest clinic in February 2023 by his general practitioner with a two-month history of persistent cough, significant fatigue, poor appetite, and unintentional weight loss since December 2022. His medical history included a recently treated lower respiratory tract infection and type 2 diabetes mellitus. He also had a history of colorectal cancer diagnosed in 2012, for which he underwent right hemicolectomy followed by adjuvant chemotherapy. He was subsequently discharged by the oncology team in 2018 following the successful completion of the treatment for colorectal malignancy.

Systemic examination and routine blood investigations were unremarkable. The patient had no known drug allergies and was taking atorvastatin, metformin, gliclazide, ramipril, alogliptin, and amlodipine. He was a retired plastic molding worker with no known history of asbestos exposure. He was a lifelong nonsmoker and remained fully independent in performing activities of daily living.

**Figure 1:**
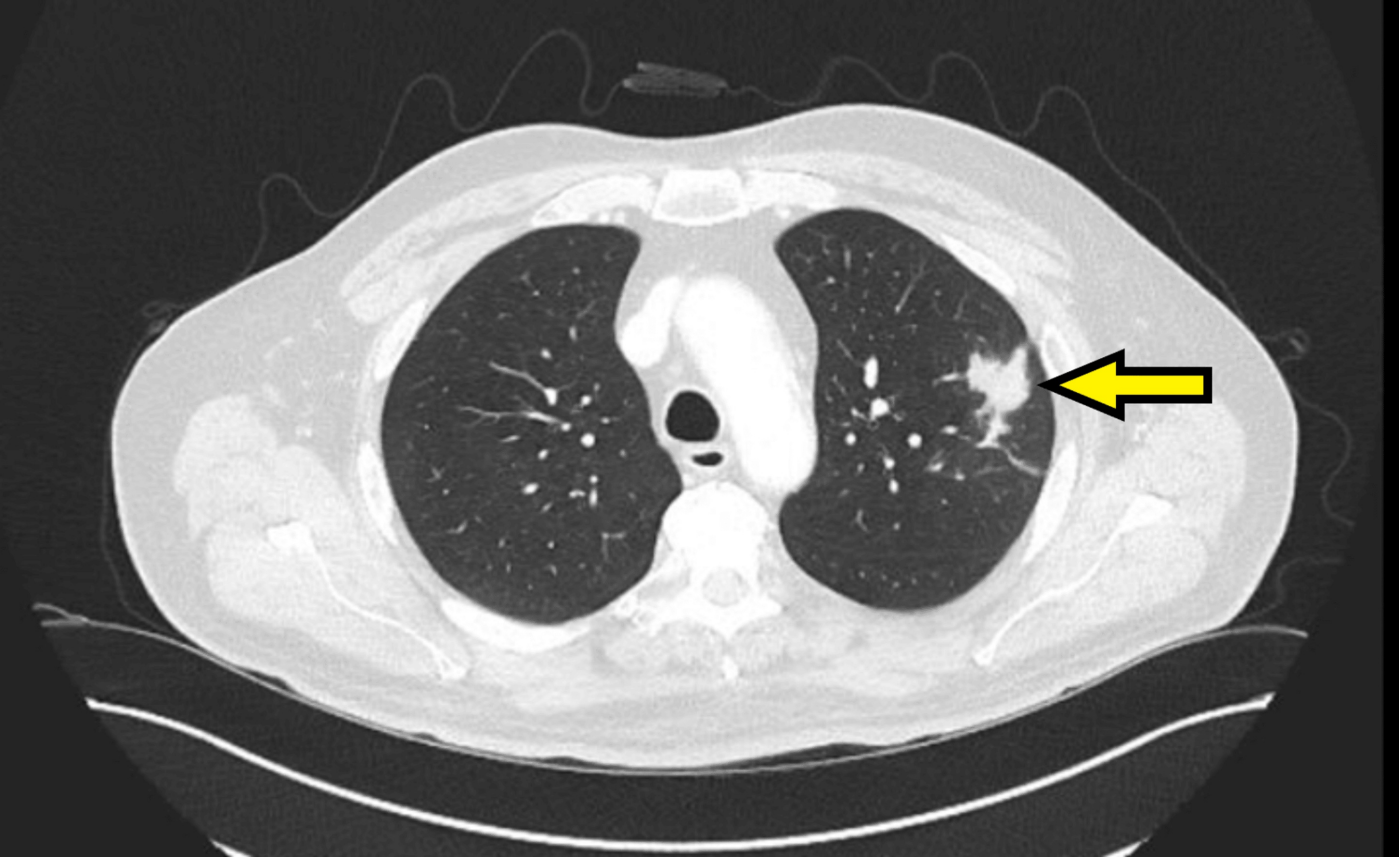
Axial CT scan of the chest demonstrating a 17 mm soft tissue nodule in the left upper lobe (yellow arrow) CT: computed tomography

**Figure 2:**
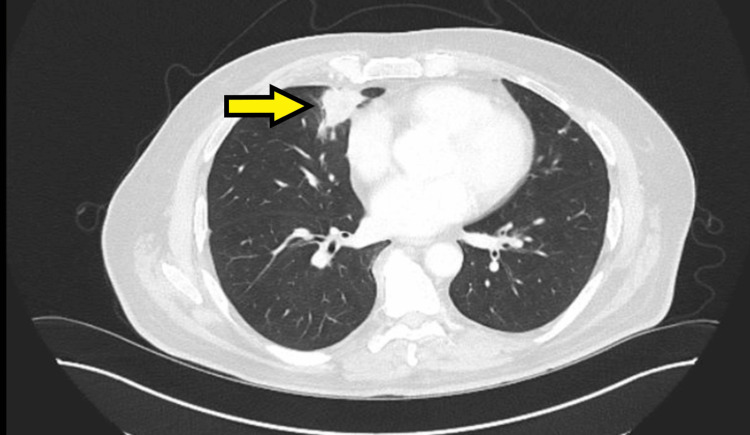
Axial CT scan of the chest demonstrating a 31 × 24 mm soft tissue density lesion in the medial segment of the right middle lobe (yellow arrow) CT: computed tomography

Computed tomography (CT) scan of the thorax, abdomen, and pelvis in February 2023 revealed two isolated pulmonary nodules: one measuring 31 × 24 mm in the right middle lobe and another measuring 17 mm in the left upper lobe, raising suspicion for either primary lung malignancy or metastatic disease (Figures 1, 2). As part of the initial diagnostic workup for suspected recurrence of metastatic colorectal malignancy, the patient underwent colonoscopy and a fecal immunochemical test; however, both investigations were inconclusive. Positron-emission tomography (PET) scan performed in March 2023 demonstrated increased fluorodeoxyglucose (FDG) uptake in both pulmonary nodules, with no abnormal FDG uptake in the colon or elsewhere. A CT-guided biopsy of the left upper lobe nodule was performed but yielded nondiagnostic results. Due to the close proximity of the right middle lobe nodule to the heart, the patient was referred to cardiothoracic surgery. He subsequently underwent video-assisted thoracoscopic surgery (VATS) with wedge resection of the right middle lobe pulmonary nodule.

**Figure 3:**
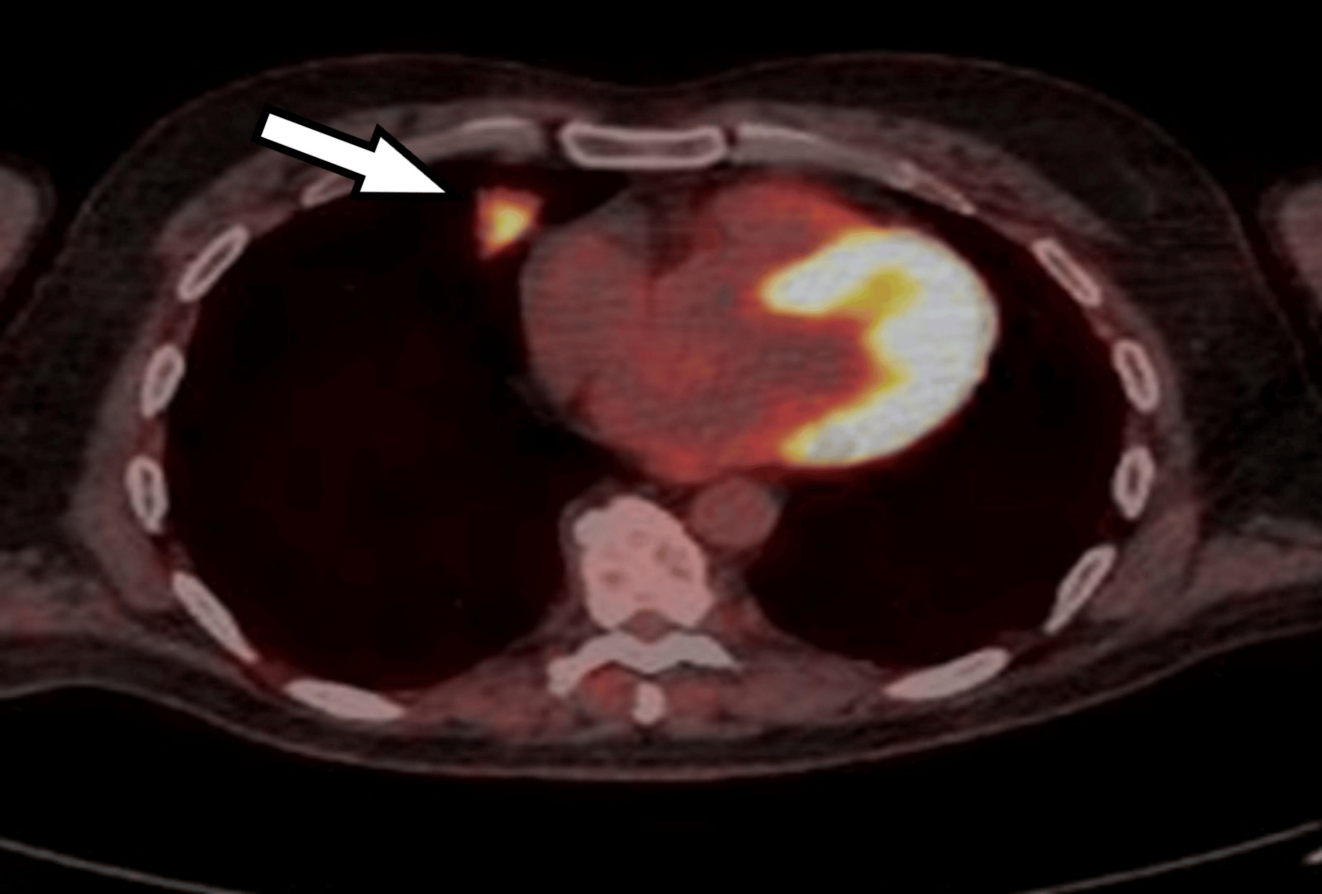
Axial PET scan of the chest demonstrating intense FDG uptake in a 31 mm soft tissue mass in the anterior aspect of the right middle lobe (white arrow) PET: positron-emission tomography; FDG: fluorodeoxyglucose

**Figure 4:**
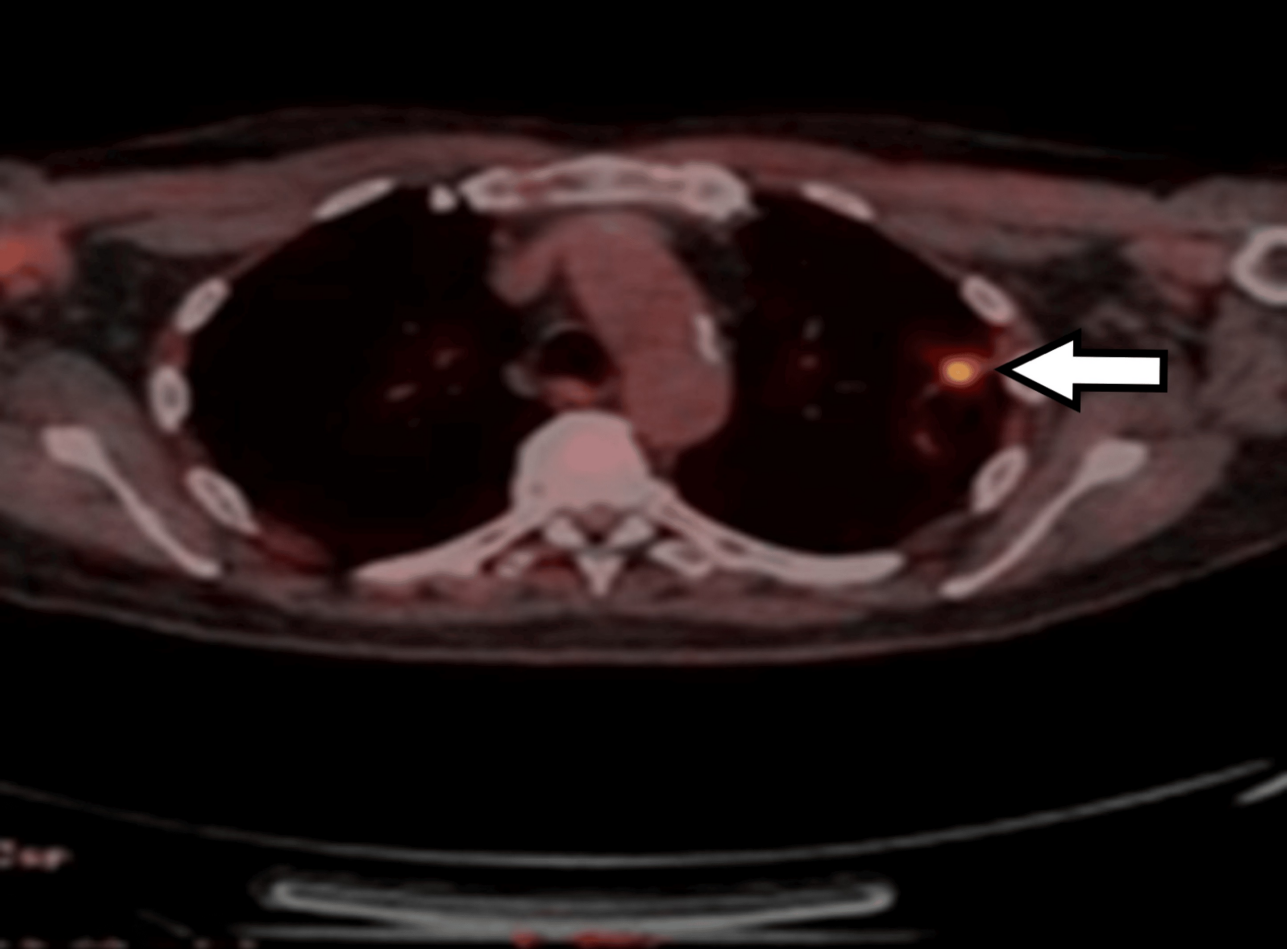
Axial PET scan of the chest demonstrating moderate FDG uptake in a 17 mm soft tissue mass with associated atelectasis in the left upper lobe (white arrow) PET: positron-emission tomography; FDG: fluorodeoxyglucose

**Figure 5:**
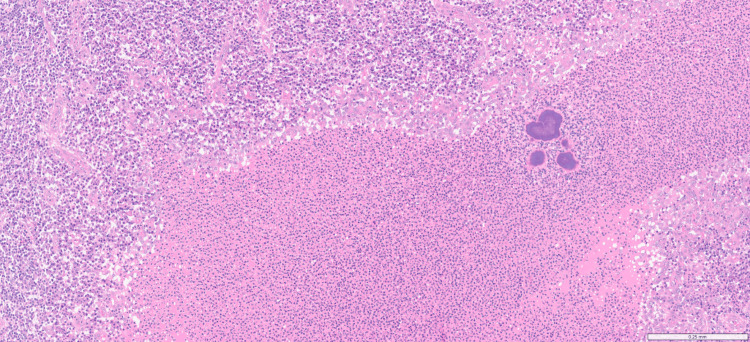
Lung tissue demonstrating one large, two medium-sized, and one small basophilic aggregate consistent with Actinomyces (H&E stain, original magnification ×100) H&E: hematoxylin and eosin

**Figure 6:**
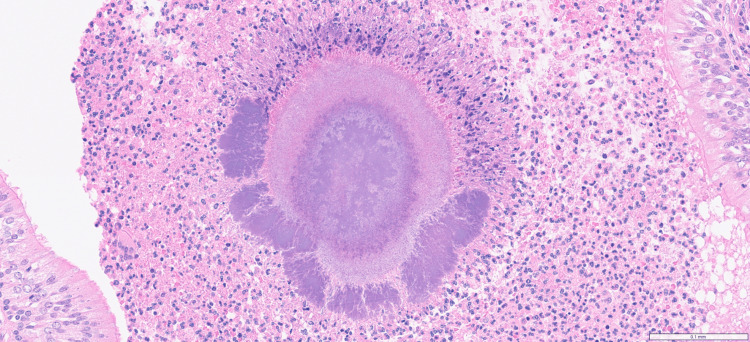
Lung tissue showing a basophilic structure consistent with Actinomyces, surrounded by dense neutrophilic infiltrates, consistent with suppurative inflammation (H&E stain, original magnification ×250) H&E: hematoxylin and eosin

Histopathological examination of the resected specimen confirmed pulmonary actinomycosis and ruled out malignancy. Microscopic evaluation revealed sections of lung tissue containing a nodule with Actinomyces associated with neutrophilic infiltration. There was no evidence of malignancy (Figures 5, 6).

**Figure 7:**
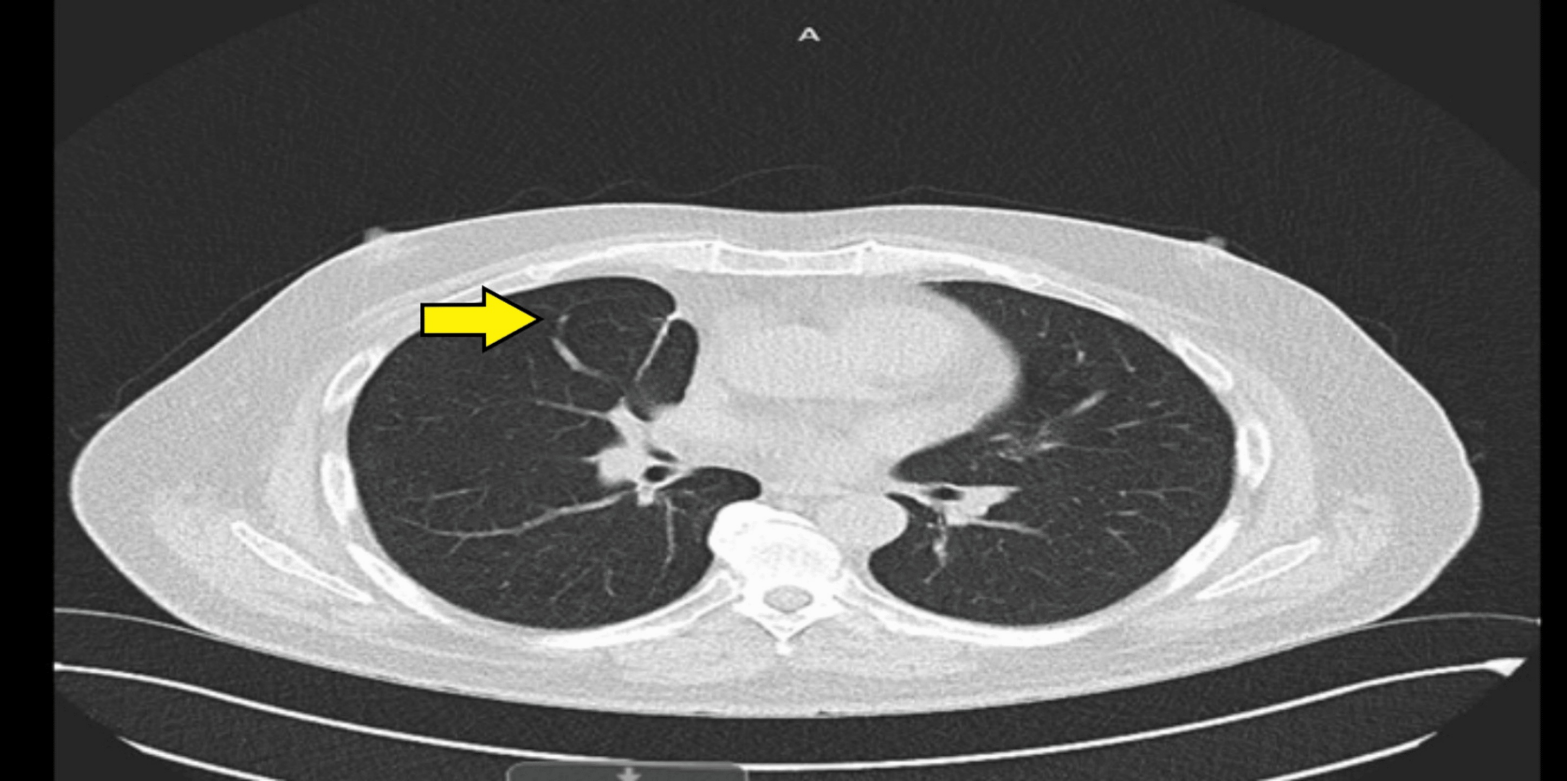
Axial CT scan of the chest demonstrating postsurgical changes in the medial segment of the right middle lobe following VATS with wedge resection (yellow arrow) CT: computed tomography; VATS: video-assisted thoracoscopic surgery

**Figure 8:**
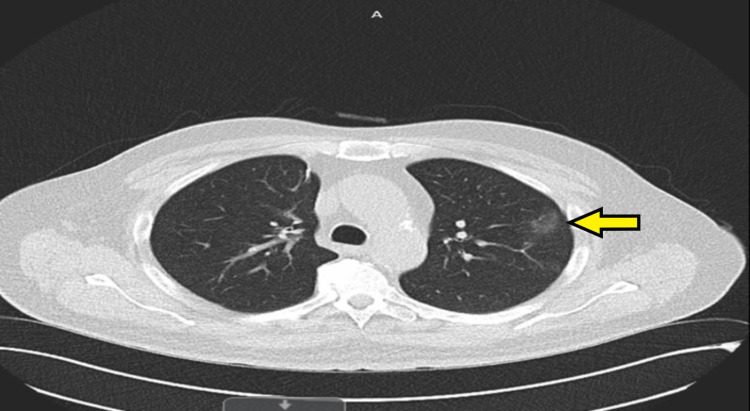
Axial CT scan of the chest demonstrating significant resolution of the left upper lobe nodule, with only a dense linear band of tissue remaining (yellow arrow) CT: computed tomography

Following the biopsy report and consultation with the infectious diseases team, the patient was initially started on intravenous ceftriaxone 2 g once daily for six weeks. However, given the patient's preference and hemodynamic stability, oral amoxicillin 1 g three times daily was initiated along with close monitoring. The patient completed a six-month course of antibiotics. At subsequent outpatient follow-up, the patient denied any constitutional or respiratory symptoms, exhibited no signs of poor oral hygiene or gastroesophageal reflux, and demonstrated appropriate weight gain. A follow-up CT scan of the chest demonstrated complete resolution of the pulmonary nodules and associated symptoms (Figures 7, 8). 

## Discussion

This case report of pulmonary actinomycosis presents a rare and complex clinical scenario that was initially investigated for suspected lung cancer. Its atypical presentation, slow progression, inconclusive PET avidity, and CT-guided biopsy results pose significant diagnostic challenges unique to this case report. This discussion explores pathophysiology, treatment options, and key difficulties in diagnosing pulmonary actinomycosis.

Pulmonary actinomycosis typically presents with nonspecific respiratory symptoms and can lead to hemoptysis in some cases if it remains untreated. Pulmonary involvement occurs in approximately 15%-20% of all actinomycosis infections. Although rare, sulfur granules in the sputum can be a characteristic feature of the infection [5].

The diagnosis is often based on clinical features, radiological findings, and identification of bacteria through histopathology. It usually presents as small nodules or cavitary lesions within the lung parenchyma, with occasional involvement of the mediastinum and pleura. Unfortunately, the role of FDG PET avidity in such cases is of limited benefit as it cannot distinguish actinomycosis from lung cancer. In such diagnostic dilemmas, a minimally invasive approach such as VATS may be reasonable for obtaining a definitive diagnosis [6]. The anaerobic nature of Actinomyces and slow growth in culture media make it difficult to isolate, increasing the risk of false-negative culture results. As a result, the diagnosis of this infection may easily be missed [7].

Despite these challenges, pulmonary actinomycosis generally carries a favorable prognosis [8]. Pulmonary actinomycosis is typically treated with a prolonged course of antibiotics for almost six months. However, some cases may require surgery if they remain unresponsive to antibiotics [9]. The drug of choice for treating actinomycosis is penicillin; however, in cases of penicillin allergy, tetracycline and erythromycin are effective alternative options. In severe, complicated cases such as discharging fistulas, empyema, or massive life-threatening hemoptysis, surgical interventions can be lifesaving [10].

## Conclusions

This case highlights the importance of considering pulmonary actinomycosis, a rare infectious etiology, in the differential diagnosis of lung nodules. This case report also highlights the diagnostic challenges associated with this condition and the critical role of imaging combined with microbiological and histopathological confirmation. While the medical management with a prolonged course of antibiotics is the mainstay of treatment, surgery can be necessary in selected cases. A multidisciplinary approach remains essential in resolving complex diagnostic uncertainties and guiding effective patient care.
